# Diverse and Abundant Secondary Metabolism Biosynthetic Gene Clusters in the Genomes of Marine Sponge Derived *Streptomyces* spp. Isolates

**DOI:** 10.3390/md16020067

**Published:** 2018-02-20

**Authors:** Stephen A. Jackson, Lisa Crossman, Eduardo L. Almeida, Lekha Menon Margassery, Jonathan Kennedy, Alan D.W. Dobson

**Affiliations:** 1 School of Microbiology, University College Cork, National University of Ireland, T12 YN60 Cork, Ireland; sjackson@ucc.ie (S.A.J.); lekha513@gmail.com (E.L.A.); edleaoalmeida@gmail.com (L.M.M.); 2 School of Biological Sciences, University of East Anglia, Norwich Research Park, Norwich NR4 7TJ, UK; L.Crossman@uea.ac.uk; 3 SequenceAnalysis.co.uk, NRP Innovation Centre, Norwich NR4 7UG, UK; 4 Invista Performance Technologies, The Wilton Centre, Wilton, Redcar, Cleveland TS10 4RF, UK; jonathan.kennedy@invista.com; 5 Environmental Research Institute, University College Cork, National University of Ireland, Lee Road, T23 XE10 Cork, Ireland

**Keywords:** marine *Streptomyces*, biosynthetic gene clusters, secondary metabolites

## Abstract

The genus *Streptomyces* produces secondary metabolic compounds that are rich in biological activity. Many of these compounds are genetically encoded by large secondary metabolism biosynthetic gene clusters (smBGCs) such as polyketide synthases (PKS) and non-ribosomal peptide synthetases (NRPS) which are modular and can be highly repetitive. Due to the repeats, these gene clusters can be difficult to resolve using short read next generation datasets and are often quite poorly predicted using standard approaches. We have sequenced the genomes of 13 *Streptomyces* spp. strains isolated from shallow water and deep-sea sponges that display antimicrobial activities against a number of clinically relevant bacterial and yeast species. Draft genomes have been assembled and smBGCs have been identified using the antiSMASH (antibiotics and Secondary Metabolite Analysis Shell) web platform. We have compared the smBGCs amongst strains in the search for novel sequences conferring the potential to produce novel bioactive secondary metabolites. The strains in this study recruit to four distinct clades within the genus *Streptomyces*. The marine strains host abundant smBGCs which encode polyketides, NRPS, siderophores, bacteriocins and lantipeptides. The deep-sea strains appear to be enriched with gene clusters encoding NRPS. Marine adaptations are evident in the sponge-derived strains which are enriched for genes involved in the biosynthesis and transport of compatible solutes and for heat-shock proteins. *Streptomyces* spp. from marine environments are a promising source of novel bioactive secondary metabolites as the abundance and diversity of smBGCs show high degrees of novelty. Sponge derived *Streptomyces* spp. isolates appear to display genomic adaptations to marine living when compared to terrestrial strains.

## 1. Introduction

Microbially derived natural products are an important source of novel biotherapeutic agents, with more than 22,000 biologically active compounds being isolated to date from microorganisms [1]. Approximately 45% of these are produced by *Actinobacteria*, with members of the genus *Streptomyces* being particularly proficient producers [2]. *Actinomycetes* have produced a number of important drug leads which have subsequently been developed into immunosuppressive (rapamycin), anti-cancer (bleomycin, daunorubicin, mitomycin) and anti-microbial (amphotericin B, erythromycin, vancomycin) drugs. However, there is an ongoing need for novel bioactive compounds, particularly antibiotics, due to the emergence of antibiotic resistance in clinically important bacterial pathogens [3]. The rapid dissemination of antibiotic resistances has coincided with the antibiotic “discovery void” where no new antibiotic scaffolds have been identified since 1987 [4].

Strategies to address the dearth of newly identified clinical therapeutics have progressed through extensive screening regimes of environmental microbial isolates [5], through the dawn of metagenomics where environmental DNA was cloned to heterologous expression hosts [6]. This promised to allow access to the genetic repertoires of uncultivable microbes but resulted in the “great screen anomaly” [7] where gene and product discovery from clone libraries is disappointingly low compared to what might have been expected. The trend has now returned to investigating cultivable isolates, armed with powerful genome sequencing technologies which have made huge numbers of draft and completely resolved bacterial genomes available for “genome mining”. The somewhat surprising outcome of these efforts is that the genomes of, for example, Actinobacteria contain dozens of secondary metabolism biosynthetic gene clusters (smBGCs), the majority of which appear to be “silent” or “cryptic” [8]. This discovery led to the estimate that no more than 10% of the genes encoding small molecules in bacteria have to date been identified and furthermore that a single gene cluster can give rise to a suite of related compounds which can act synergistically to inhibit bacterial growth [9]. Further conservative estimates derived from genome sequence data suggest that the genus *Streptomyces* alone may be capable of producing 150,000 secondary metabolites with fewer than 5% of these having been characterized to date [10].

These findings have focused efforts to attempt to activate these silent clusters through imaginative approaches such as OSMAC (one strain many compounds) cultivation efforts, co-cultivation of microbes to provide environmental cues or stresses, using genome data to ‘awaken’ gene cluster activators, to delete suppressors or to engineer strong promoters upstream of BGCs of interest [11]. A high-throughput approach, termed high-throughput elicitor screens (HiTES), has been developed where reporter genes such as *lacZ* or GFP are cloned into a BGC of interest and the strain is exposed to libraries of small compound potential ‘elicitors’ such as sub-inhibitory concentrations of antibiotics [12]. The elegance of this approach is that endogenous regulation of gene expression is maintained, the reporter genes allow the identification of the previously silent clusters, the gene cluster activator is identified, and the small molecule gene cluster product is also identified. This approach allowed Xu and colleagues to identify two cytotoxins as inducers of BGCs in *Streptomyces albus* J1074, and to characterize 14 novel small molecule products, one of which is a novel antifungal and another which may find use as an anti-cancer agent [13].

Genome mining of terrestrial actinomycete bacteria has proven to be useful in the identification of secondary metabolism biosynthetic gene clusters (smBGCs) such as those encoding the bottromycin, planosporicin and tunicamycin clusters [14] and it is clear that based on the successful genome mining on the *Salinispora* genus, which resulted in the identification of a number of novel biosynthetic gene clusters [15], and the recent work on the anthracimycin gene cluster from the marine derived *Streptomyces* sp. T676 [16] amongst others, that genome mining may also prove effective in the identification of potentially novel chemistry from other marine derived actinomycete bacteria.

Marine derived actinomycetes are known to produce a broad range of structurally complex secondary metabolites with broad biological activities, and they are a source of an ever increasing number of natural products [17,18]. Novel *Actinomycetes,* including novel *Streptomycetes*, have also been isolated from deep-sea environments. The deep-sea is a harsh environment with high pressures, low temperatures, high salinity and low light and oxygen concentrations [19]. Interestingly, *Streptomycete*-derived novel bioactive secondary metabolites have been identified from isolates from the deep-sea [19]. Antibacterial and cytotoxic metabolites, are produced from a type I PKS gene cluster by *Streptomyces* sp. SCSIO 01127 which was isolated from a depth of 1350 m in the South China Sea [20] while antibacterial compounds, are produced by *Streptomyces scopuliridis* SCSIO ZJ46 which was isolated from a depth of 3536 m in the South China Sea [21]. *Streptomyces drowzdowiczii* SCSIO 10141, isolated from 1396 m in the South China Sea is known to produce the anti-infective metabolites [22]; cytotoxic and antibacterial compounds are produced by another deep sea isolate, *Streptomyces niveus* SCSIO 3406 [23].

With this in mind and following an extensive screening regime of marine actinomycete bacteria which we had previously isolated from the shallow water sponge *Haliclona simulans* [24] and from two deep-sea sponges *Lissodendoryx diversichela* and *Stelletta normanii* [25], involving growth inhibition of a number of clinically relevant bacterial and fungal species, 13 marine sponge-derived *Streptomyces* spp. strains were chosen for whole genome sequencing in an attempt to determine the secondary metabolite biosynthetic potential of these strains.

## 2. Results and Discussion

### 2.1. Antimicrobial Activities

A total of over 540 actinomycetes, including some which had previously been isolated from shallow water and deep-sea sponges in Irish waters [24,25,26], were screened for growth inhibition of a number of clinically relevant bacterial and fungal/yeast species. Thirteen of these strains which displayed the most interesting range of bioactive antimicrobial activities, including growth inhibition of problematic anti-microbial resistant (AMR) human pathogens such as methicillin-resistant *Staphylococcus aureus* (MRSA) and vancomycin-intermediate *Staphylococcus aureus* (ViSA), were identified for subsequent analysis (Table 1).

### 2.2. Genome Sequencing

The genomes of these 13 *Streptomyces* spp. were sequenced using Illumina MiSeq paired-end sequencing. These strains were designated as follows: SM1, SM5, SM9, SM10, SM11, SM12, SM14, SM16, SM17, SM18, FMC008 isolated from shallow water sponges and B188M101 and B226SN101, isolated from deep-sea sponges. Genome assemblies resulted in a large number of contigs (*n* = 195–1592) (Table 2). Gap closure of these assemblies was hampered by the typical high GC content of the genomes of *Streptomyces* spp., as well as the presence of very many highly repetitive sequences in gene clusters such as polyketide synthase (PKS) and nonribosomal peptide synthetases (NRPS) clusters. The genomes ranged in size from 6.41 to 8.44 Mb (Table 2).

### 2.3. Taxonomy and Phylogeny

**Figure 1:**
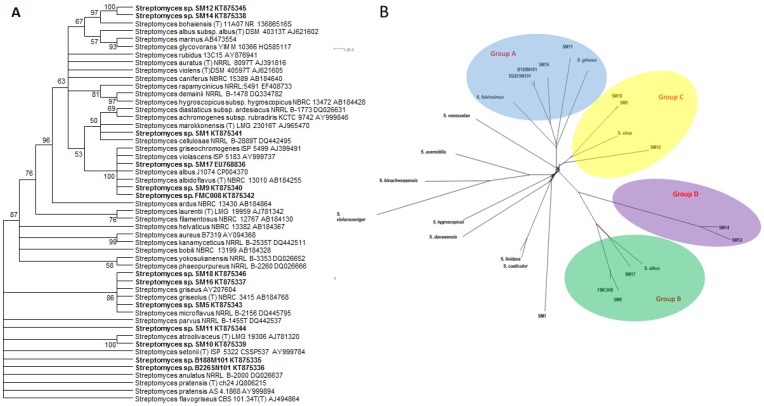
(**A**) 16S rRNA gene based (*Streptomyces* isolates from this study are in bold font) and (**B**) whole genome Frequency feature profile based phylogenetic trees of *Streptomyces* spp. marine isolates.

The phylogeny of the 13 marine *Streptomyces* was determined by analysis of the 16S rRNA genes (Figure 1A), and by Feature Frequency Profile (Ffp) [27] of the whole genomes (Figure 1B), together with genomic analysis using Kraken (Supplementary Table S1) [28]. In the latter analysis, greater than 75% of the sequence reads were assigned closely to a known species while three of the strains (SM1, SM12 and SM14) could only be assigned at the genus level (Table 3). 

The strains recruited to four main groups, Group A (comprising *S. griseus*, *S. fulvissimus* as well as SM11 and SM16 and both of the deep sea strains, B188M101 and B226SN101), Group B (comprising *S. albus*, SM17, SM9 and FMC008), Group C (comprising *S. sirex*, SM18, SM5 and SM10) and Group D (comprising SM12 and SM14) (Figure 1B). Analysis of the 16S rRNA genes suggest that SM1 is closely related to the group D isolates in a polyphyletic clade which is a sister clade to the Group B isolates, however Ffp based phylogeny indicates that SM1 falls outside of that polyphyletic clade.

### 2.4. Pan Genome and Core Genome

**Figure 2:**
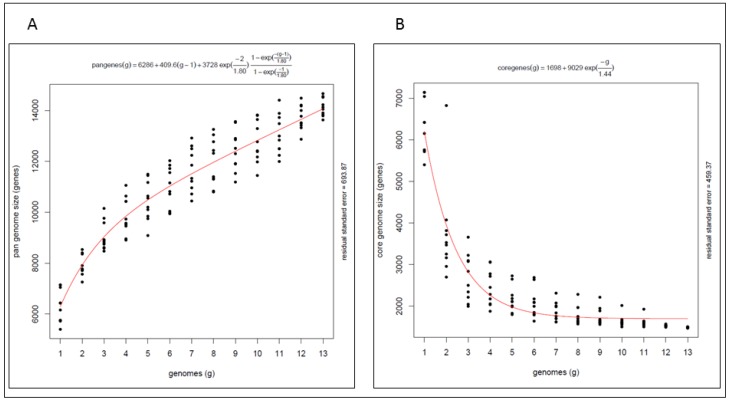
(**A**) Pangenome and (**B**) core genome of *Streptomyces* spp. marine isolates.

The pan genome (Figure 2A) of these marine isolates is open with an average of 648 genes added for each additional genome considered and a total gene complement of 14,066 genes shared amongst the isolates. The core genome (Figure 2B) comprises 1699 genes indicating that only 12% of the pan genome is shared by all of the strains examined here. The pan genome analysis highlights the genetic diversity within the genus, indicating that the biosynthetic potential of these marine *Streptomyces* warrants further investigation, including the potential for biodiscovery of novel secondary metabolites from these isolates. 

The highest intra-group sequence conservation, and therefore the lowest diversity, was seen within the *Streptomyces albus* group (Group B—SM9, SM17 and FMC008), suggesting that this group may prove to be less interesting in terms of potential discovery of novel bioactive secondary metabolites. While this hypothesis is supported by antiSMASH [29], analyses of the genomes of strains SM9 and FMC008 (which noticeably harbours the lowest abundance of smBGCs of any of the strains in this study—16 and 15 clusters, respectively) but not when considering the genome of strain SM17 (comprising 49 secondary metabolism gene clusters—the fourth highest abundance of any of the strains in this study).

### 2.5. Secondary Metabolism Biosynthetic Gene Clusters

The 13 *Streptomyces* spp. genomes were investigated for the presence of smBGCs of potential interest. All of the genomes examined contained numerous smBGCs as identified by antiSMASH (Table 3; Supplementary Tables S2–S14). The 13 strains contain a combined total of 485 individual clusters which share homology to 87 distinct known gene clusters whose metabolic product is known. While some clusters were common to many strains, all strains also contained clusters unique to that strain only (48 gene clusters are found in only one strain). The most commonly shared clusters were those showing similarities to the known clusters which produce the compatible solute ectoine (11 strains), the siderophore desferrioxamine B and the anti-tumour metabolite herboxidiene (7 strains), the peptide siderophore coelichelin, the carotenoid light-harvesting pigment isorenieratene and the terpene hopene (6 strains). The abundances or types of particular smBGCs are not a clear indicator of phylogenetic relatedness or of the isolation source (host sponge or sampling depth).

Group A isolates display the largest genome sizes (Table 1) and with the exception of SM16 (*n* = 39) also possess the most abundant smBGCs (*n* = 51–54). The genomes of the Group A isolates show many similarities with all strains harbouring gene clusters related to the known clusters which produce the previously characterised metabolites ectoine, desferrioxamine B, coelichelin, herboxidiene and isorenieratene and also for γ-butyrolactone, alkylresorcinol and griseobactin. While these latter three metabolites are shared by all Group A strains, they are also exclusive to that group. Three of the four Group A strains also harbour gene clusters with similarities to the cluster known to produce the antibiotic friulimicin produced by the actinomycete *Actinoplanes friuliensis*, daptomycin, the heat-stable antifungal factor (HSAF) and also the peptide morphogen AmfS. 

Gene clusters unique to one Group A isolate include those related to the production of thaxtomin, skyllamycin, balhimycin, griseoviridin, kanamycin, surfactin, myxothiazol A, pristinamycin, streptolydigin, concanamycin, chartreusin, naringenin, platensimycin, kosinostatin, oxazolomycin, bacillibactin, neocarzinostatin, glycolipopeptide, guadinomine and borrelidin.

Despite the phylogenetic relatedness of Group A isolates, the bioactivity profiles of the strains were not consistent. The deep-sea strains (B188M101, B226SN101) only displayed activity against yeasts while those sourced from shallow waters were only active against bacteria. Both Group A shallow water isolates (SM11, SM16) inhibited the growth of problematic drug resistant *Staphylococcus* spp. (Table 1). This makes further investigation of the smBGCs of these strains of high interest.

Groups B, C and D strains are less similar with few gene clusters shared by all members of any of those groups, except for ectoine biosynthesis genes which are highly similar in all Group B and Group C strains. 

Two of the Group B strains (SM9, FMC008) harbour the fewest smBGCs (Table 3) in this study while the genome of the other Group B strain (SM17) is noticeably enriched in NRPS gene clusters. Analysis by antiSMASH shows that 15 of the 18 NRPS clusters in the SM17 genome show no similarities to known NRPS gene clusters. Analysis of Pfam domains of these clusters condensation (Supplementary Figures S1–S3) and epimerization domains (Supplementary Figure S5A) are diverse and abundant and form clades which correlate closely with phylogeny and show high similarities to those domains in the genome of *S. albus* J1074. All Group B strains harbour gene clusters with low similarity to the antimycin production cluster from *S. albus* J1074. Two of the three Group B strains also harbor clusters with low similarities to complestatin biosynthesis genes (SM9 and SM17) from *S. albus* J1074, the desotamide production cluster (SM9 and FMC008) from *Myxococcus fulvus* HW-1 and mannopeptimycin production genes (SM9 and FMC008) from *Streptomyces hygroscopicus*. The abundance of unknown NRPS gene clusters in the genome of SM17 as well as its broad range of antimicrobial activities versus *E. coli* and MRSA (Table 1) warrant further research efforts aimed at identifying the encoded metabolites.

Two of the three Group C strains contain gene clusters showing relatedness to the clusters known to produce mirubactin and paenibactin in *Streptomyces* sp. NTK 937, coelibactin in *Streptomyces lividans* TK24 and bafilomycin in *Streptomyces lohii* strain ATCC BAA-1276 (SM5 and SM18), together with cystothiazol A in *Streptomyces griseus* subsp. *griseus* NBRC 13350 and steffimycin in *Streptomyces fulvissimus* DSM 40593 (SM10 and SM18). This group contains numerous PKS, NRPS and PKS/NRPS hybrid clusters and display antibiotic activity against *E. coli* (SM5 and SM10) and against hVISA (SM10) and MRSA (SM18) while also inhibiting the growth of *Bacillus* spp. (Table 1). Thirteen of the smBGCs in the genome of SM18, including PKS, NRPS, PKS/NRPS hybrid, lantipeptide and bacteriocin clusters show no relatedness to known clusters in antiSMASH analysis. 

The only secondary metabolism gene cluster common to Group D isolates displays low levels of similarity to the cluster which produces zorbamycin in *Streptomyces flavoviridis* ATCC 21892. Both Group D strains (SM12 and SM14) inhibited the growth of hVISA (Table 1) making them of particular interest because they appear phylogenetically distant from all other strains presented here. The strains could not be assigned to a genus in Kraken analysis (Supplementary Table S1) and appear phylogenetically distant in FFP analysis (Figure 1B). The genome of SM12 hosts 18 Type I PKS clusters of which 10 share no homology with known gene clusters in the linked Minimum Information about a Biosynthetic Gene Cluster (MIBiG) database (https://mibig.secondarymetabolites.org/index.html) [30]. Increasing the taxonomic diversity of available *Streptomyces* spp. strains available for screening regimes is predicted to increase the chemical diversity of identified metabolites. With that in mind, these phylogenetically remote strains with demonstrated bioactivities and numerous uncharacterised PKS gene cluster products are of high interest.

The genome of SM1, which does not recruit to the groups (A–D) described here, also harbours a unique secondary metabolism gene cluster profile. While SM1 does harbour clusters for the frequently observed desferrioxamine B and isorenieratene production genes, it is one of only two genomes presented here not to encode ectoine biosynthesis genes. Of the 28 gene clusters in the genome of SM1, as identified by antiSMASH, only nine of those clusters show homology to known gene clusters and three of these are unique to SM1 in our study. As this strain has displayed broad-range antimicrobial activity and appears only distantly related to other strains in Figure 1B and distantly related to the metabolically talented *S. coelicolor* A3(2) in Kraken analysis (Supplementary Table S1), it also warrants further investigation with respect to the further characterization of its metabolites.

### 2.6. Protein Family (Pfam) Domain Analysis

**Figure 3:**
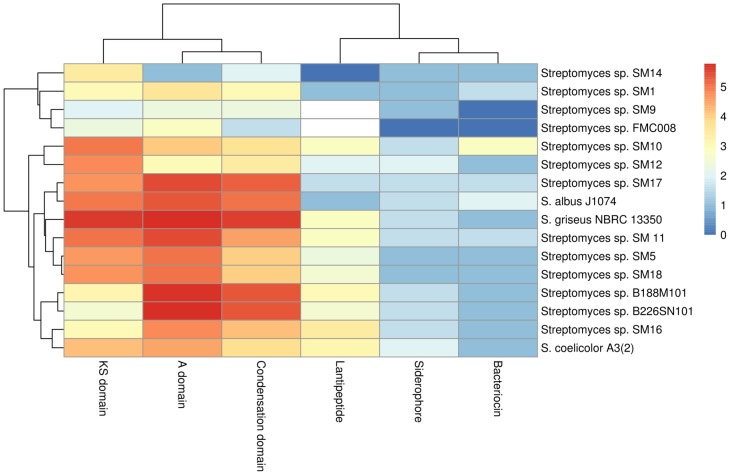
Log2 heatmap of predicted secondary metabolism protein domains of interest from marine *Streptomyces* spp. isolates and from select reference terrestrial genomes.

The genome assemblies of the 13 marine *Streptomyces* strains were highly fragmented, and so smBGC prediction can be hampered by particular clusters being fragmented across different contigs. This can lead to an over-prediction of the numbers of clusters present. Nonetheless it was possible to analyse deduced proteins at the domain level. Translated sequences of protein domains of interest: ketosynthase (KS) domains of PKS genes (Supplementary Figure S1), condensation starter domains (Supplementary Figure S2), condensation DCL domains (Supplementary Figure S3), condensation LCL domains (Supplementary Figure S4) epimerization (Supplementary Figure S5A) and heterocyclization (Supplementary Figure S5B) domains of NRPS gene clusters, IucA-IucC domains of siderophore genes (Supplementary Figure S6), DUF692 domains of bacteriocin genes (Supplementary Figure S7) and LanC domains of lantibiotic genes (Supplementary Figure S8) were extracted from the antiSMASH results using a custom script. These domains were analysed and are presented as a heat-map (Figure 3) with the evolutionary relationships of these protein domains shown in phylogenetic trees (Supplementary Figures S1–S8).

#### 2.6.1. KS Domains of PKS Gene Clusters 

Of 25 KS domain sub-clades identified (Supplementary Figure S1), 12 of these clades include only one of the phylogenetic sub-groups (A–D) described earlier (Figure 1B), suggesting a link between the phylogeny of the isolates and a subset of the KS domains therein. As the remaining 13 clades of KS domains contain domains from two or more phylogenetic subgroups, it appears that these KS domains are not phylogeny-related but represent a more general genetic diversity. It is clear from the tree (Supplementary Figure S1) that the number of clades and the individual branch lengths, indicate that very high levels of diversity are apparent even within a single protein domain type amongst a limited number of isolates from this single genus.

#### 2.6.2. Nrps Gene Clusters

Protein domains from NRPS clusters (condensation starter, condensation DCL, condensation LCL, epimerization and heterocyclization were analysed individually by sequence alignments and phylogenetic tree building (Supplementary Figures S2–S5). Thirteen distinct clades of condensation starter domains were observed (Supplementary Figure S2). These domains appear diverse but are more closely distinguishable as recruiting to phylogenetic groups A–D (Figure 1B) than is observed in the KS domain analysis (Supplementary Figure S1). Six of the clades contain domains from only one phylogenetic sub-group while one of these clades includes domains from all four phylogenetic sub-groups. It is noted that a single isolate may contain more than one condensation starter domain gene (where more than one NRPS gene cluster is present). These condensation starter domains may be highly similar (e.g., condensation starter domains of SM9— Supplementary Figure S2) or indeed very different (e.g., condensation starter domains of SM12—Figure S2). It is also noteworthy that although the genome of SM14 harbours two NRPS gene clusters, no condensation starter domain has been identified in these clusters. When considering other NRPS domains, condensation DCL (Supplementary Figure S3) and condensation LCL (Supplementary Figure S4), epimerization (Supplementary Figure S5A) and heterocyclization (Supplementary Figure S5B) domains, many phylogenetic clades harbour sequences which correlate with the taxonomic phylogeny described in Figure 1B. As before, no epimerization or heterocyclization domains were identified in the NRPS clusters of Group D strains (SM12 and SM14). Nonetheless, even within the clades identified, noticeable degrees of diversity are apparent (i) within strains with multiple gene clusters, (ii) within phylogenetic clades and (iii) overall. The tree topologies and branch lengths indicate high levels of gene diversity in marine *Streptomyces* spp. Group A isolates (SM11, SM16, B188M101 and B226SN101) appear enriched for adenylation (Figure 3) and condensation (Supplementary Figures S2–S4) domains of NRPS gene clusters when compared to the other phylogenetic sub-groups described here. There appears to be a stronger link between taxonomic phylogeny and the evolutionary phylogeny of NRPS epimerization (Supplementary Figure S5A) and NRPS heterocyclization domains (Supplementary Figure S5B). These domains are highly abundant in Group A and Group B isolates but entirely absent from the genomes of Group D strains. The majority of predicted protein domains from the deep sea isolates (B188M101 and B226SN101) are more similar to each other than to similar genes in shallow water or terrestrial isolates. This supports previous findings by our group where we reported putative deep sea sponge specific microbiome [24] and secondary metabolome [31].

#### 2.6.3. Siderophores, Bacteriocins and Lantibiotics

Phylogeny-related patterns of predicted protein domains of siderophores (IucA-IucC— Supplementary Figure S6), bacteriocins (DUF692—Supplementary Figure S7) and lantipeptides (LanC-like—Supplementary Figure S8) were notable with the majority of those domains identified here, clustering more closely to domains from within the phylogenetic subgroups than to those of other subgroups. Bioavailable iron in ocean waters has long been recognized as a limiting factor for growth [32]. Dissolved iron concentrations are orders of magnitude higher in surface and mesopelagic waters when compared to the deep sea [33]. Thus, it might be expected that microbes from deep waters may contain more genes involved in iron chelation or with higher degrees of siderophore gene diversity. We have not observed this however and possible explanations may be that the genes in the deep sea strains are more highly transcribed than those of strains from shallower waters, or that the symbiotic relationship between sponges and their resident microbiota may provide higher concentrations of iron to the microbes than is available to planktonic ocean microorganisms. Iron has been identified as an important mineral for sponge primmorph proliferation and morphogenesis [34] and it has been demonstrated that some sponges can accumulate trace elements, including iron, at concentrations high above those of seawater and sediments [35].

Eleven of the 13 genomes described here host DUF692 domains found in bacteriocin production genes, the exceptions being SM9 and FMC008 despite antiSMASH predictions of one bacteriocin gene cluster in each of those genomes. Conversely, the genome of SM14 hosts a DUF692 domain, but no bacteriocin cluster was predicted for this strain by antiSMASH. Although five NRPS gene clusters were predicted in the genome of SM10 by antiSMASH, only three DUF692 domains were identified. Nonetheless, two of those domains are notably different to all other DUF692 domains identified here and to each other. The genomes of all other strains host one DUF692 gene copy each. Degrees of conservation in all deduced protein sequences apart from the aforementioned SM10 sequences are evident from the phylogenetic tree (Supplementary Figure S7), though an evolutionary pattern correlated with taxonomic phylogeny can also be seen.

When considering the lantibiotic (LanC-like) domains, Groups A (SM11, SM16, B188M101 and B226SN101) and C (SM5, SM10 and SM18) as well as the terrestrial strains *S. griseus* and *S. coelicolor*, hosted more potential LanC-like domains when compared to Groups B (SM9, SM17 and FMC008) and D (SM12 and SM14) (Supplementary Figure S8). Strains B188M101 and SM16 hosted the most lantibiotic smBGCs in this study (four and five respectively) and also hosted the most LanC-like protein domain gene sequences (five and seven respectively). All seven of these protein domains on the SM16 genome are quite different to each other indicating that diverse lantipeptides may potentially be produced by that strain.

### 2.7. Marine Adaptations

**Figure 4:**
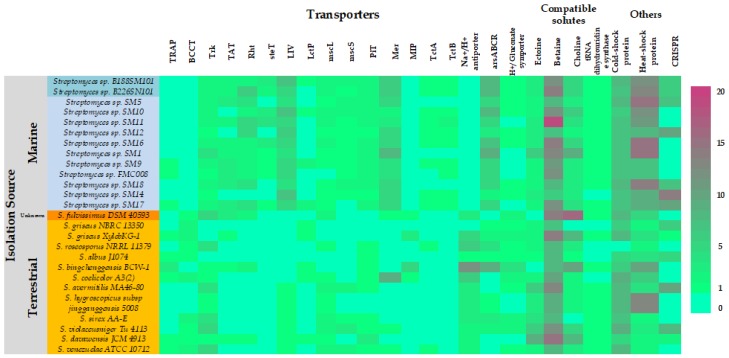
Marine adaptations in *Streptomyces* spp.: Abundances of compatible solutes and osmoprotectant biosynthesis and/or transport genes and of CRISPR genes in the genomes of marine *Streptomyces* spp. isolates (blue background) and reference terrestrial *Streptomyces* spp. genomes (orange background). TRAP: Tripartite ATP-dependent Periplasmic transporters (organic acid import); BCCT: Betaine Carnitine Choline Transporter; Trk: Potassium ion transport; TAT: Twin Arginine Translocator; Rht: homoserine/threonine transport; steT: serine/ threonine exchanger transporter; LIV: Transport of Branched-Chain Amino Acids; LctP: lactose permease; mscL: Large Conductance Mechanosensitive Ion Channel; mscS: Small Conductance Mechanosensitive Ion Channel; PiT: Sodium-dependent phosphate transporter; Mer: Mercury transporter; MIP: major intrinsic protein; TctA: Tripartite tricarboxylate transporter; TctB: Tripartite tricarboxylate transporter; arsABCR: Arsenite detoxification system.

The 13 marine *Streptomyces* genomes were interrogated for the presence of genes that may be potentially involved in the biosynthesis and/or transport of compatible solutes and other osmoregulatory systems, which have previously been suggested to play a role in marine adaptation of microorganisms [36]. The abundances of these genes were compared to abundances in the terrestrial *Streptomyces* spp. genomes which were used to construct the Ffp phylogenetic tree (Figure 4).

The marine isolates appear to be enriched for mercury and arsenic transport systems, branched-chain amino acid transport (LIV), betaine and choline biosynthesis and heat shock proteins. The marine strains appeared however to be deficient in sodium-proton antiporters, tripartite ATP independent periplasmic transporters (TRAP), betaine transport (BCCT), serine/threonine export (Rht), aquaporins (MIP) transport and cold shock proteins. It is interesting that the genomes of the marine strains are enriched for the arsenic transport genes in light of the recent finding that sponge associated *Entotheonella* spp. sequester arsenic in intracellular vesicles whilst residing in its sponge host, *Theonella swinhoei* [37]. More work will be necessary to determine if the *Streptomyces* strains described here are preferentially exporting arsenic or importing and sequestering it as a host-protection symbiotic function. Our analysis also suggests that betaine may be the preferred compatible solute amongst marine *Streptomyces* spp. as indicated by the higher abundances of betaine biosynthetic genes when compared to those of ectoine or choline. This however needs to be experimentally confirmed by assessing transcription rates of those genes.

## 3. Materials and Methods 

### 3.1. Sponge Sampling 

The marine sponge *Haliclona simulans* (class *Demospongiae*, order *Haplosclerida*, family *Chalinidae*) was sampled by SCUBA diving at a depth of 15 m in Kilkieran Bay, Galway, Ireland (N 53°18′56.6′′, W 09°40′08.4′′) [24]. The sponge *Lissodendoryx diversichela* (class *Demospongiae*, order *Poecilosclerida*, family *Coelosphaeridae*) was sampled using the ROV *Holland I* from the *RV Celtic Explorer* at a depth of 1350 m in the North Atlantic Ocean (N 54°21′25.2′′, W 12°14′49.9′′) off the west coast of Ireland [25]. The sponge *Inflatella pellicula* (class *Demospongiae*; order *Poecilosclerida*; suborder *Myxillina*; family *Coelosphaeridae*) was sampled using the ROV *Holland I* at a depth of 2900 m in the North Atlantic Ocean (N 54°03′28.8′′, W 12°32′53.5′′) [26].

### 3.2. Culture Isolation

Sponge samples were rinsed with sterile artificial seawater (3.33% Instant Ocean™—Aquatic Eco-Systems Inc., Apopka, FL, USA) to remove exogenous materials. Sponge tissues (~1 g) were macerated with a sterile razor blade. Serial dilutions of the macerated tissues were spread to 3 isolation media; (i) starch-yeast-peptone seawater agar (SYP-SW): 1% (*w*/*v*) starch, 0.4% (*w*/*v*) yeast extract, 0.2% (*w*/*v*) peptone, 3.33% (*w*/*v*) artificial sea salts (Instant Ocean™), 1.5% (*w*/*v*) agar; (ii) modified marine agar (MMA): 0.005% (*w*/*v*) yeast extract, 0.05% (*w*/*v*) tryptone, 0.01% (*w*/*v*) β-glycerol phosphate disodium salt, pentahydrate, 3.33% (*w*/*v*) artificial sea salts (Instant Ocean™, Apopka, FL, USA), 1.5% (*w*/*v*) agar and (iii) chitin agar: 4% (*v*/*v*) colloidal chitin, 1.5% (*w*/*v*) agar. Culture plates were incubated at 28 °C for up to 8 weeks. Colonies were picked from isolation plates, streaked to fresh agar plates to obtain pure, isolated colonies. Isolates were archived by growing in liquid broth (SYP-SW) supplemented with 15% (*v*/*v*) glycerol and frozen at −80 °C. 

### 3.3. DNA Extraction

Isolates were grown overnight (~16 h) in 5 mL liquid cultures. Cells were pelleted by centrifugation (6000 g), supernatants were decanted and discarded. Cell pellets were resuspended in 467 μL TE buffer. 30 μL of 10% SDS and 3 μL of 20 mg/mL Proteinase K (Fermentas, Sankt Leon-Rot, Germany) was added to each tube and incubated for 1 h at 37 °C. An equal volume of phenol/chloroform (phenol-chloroform-isoamyl alcohol mixture ratio 25:24:1, Sigma Aldrich, Arklow, Ireland) was added and mixed well. Tubes were centrifuged, at ~18,042 g for 10 min (Eppendorf Centrifuge, 5417r, Eppendorf UK Ltd., Stevenage, UK). The upper phase of each Eppendorf tube was aspirated to fresh 2 mL Eppendorf tubes, avoiding the interphase. 100 μL of 3M sodium acetate (NaOAc) pH 5.2 was added to each tube and mixed well. 600 μL of isopropanol (Sigma Aldrich, Arklow, Ireland) was added, mixed well, and incubated at room temperature for 15 min. Tubes were then centrifuged at ~18,042 g for 20 min. Supernatants were removed and discarded. DNA pellets were washed with cold (4 °C) 70% EtOH. Tubes were centrifuged at ~18,042 g for 10 min. Ethanol was removed and discarded and DNA pellets were allowed to air-dry. DNA was re-suspended in 1 mL TE buffer. 1 μL of RNase A was added to the tubes, which were then incubated at 37 °C for 30 min. DNA was again purified by phenol extraction. DNA was analysed by gel electrophoresis and quantified using a spectrophotometer (NanoDrop ND-1000, Thermo Scientific, Gloucester, UK). The DNA solutions were stored at −20 °C.

### 3.4. 16S rRNA Gene Sequencing

16S rRNA genes were amplified by PCR using a Peltier Thermal Cycler PTC-200 PCR System (MJ Research Inc., Waltham, MA, USA). Each PCR reaction comprised 1X reaction buffer, 0.2 mM dNTPs, 0.5 μM forward primer [27f (5′-AGAGTTTGATCMTGGCTCAG-3′)], 0.5 μM reverse primer [1492r (5′-TACGGYTACCTTGTTACGACTT -3′)] [38], 1 U Taq polymerase (5 U/μL), 1.0 μL template DNA (1–10 ng), sdH2O. PCR cycle conditions comprised initial denaturation at 95 °C for 3 min followed by 30 cycles of denaturation at 95 °C for 1 min, primer annealing at 50 °C for 1 min, extension at 72 °C for 1 min followed by a final extension at 72 °C for 5 min. PCR products were analysed by electrophoresis on 1% agarose gels. PCR amplicons were sequenced by capillary electrophoresis, single extension sequencing (Macrogen Inc., Korea), using 3730xl DNA Analyser.

### 3.5. 16S rRNA Based Phylogenetic Analysis

Sequences were manually edited for quality using FinchTV 1.4.0 (Geospiza, Inc.; Seattle, WA, USA; http://www.geospiza.com). Sequence alignment and tree construction were performed using MEGA version 6 [39] (Arizona State University & Pennsylvania State University, USA, http://www.megasoftware.net/). Alignment was performed with ClustalW [40] using the Maximum Parsimony approach [41] and included bootstrap tests (*n* = 500). The evolutionary distances are in the units of the number of base substitutions per site. All positions containing gaps and missing data were eliminated from the datasets (complete deletion option). Reference sequences were downloaded from the Ribosomal Database Project (release 11, update 4, 26 May 2015, https://rdp.cme.msu.edu/) [42].

### 3.6. Bioactivity Screening

Isolates were screened for antibacterial activities against a panel of clinically relevant Gram negative [*Escherichia coli* NCIMB 12210 and *Pseudomonas aeruginosa* PAO1], Gram positive [*Bacillus* spp.—*B. subtilis* 1E32, *B. subtilis* 1A40, *B. cereus* FPL1); *Staphylococcus* spp. (hVISA [Heterogonous Vancomycin Intermediate *Staphylococcus aureus* 22900], MRSA [Methicillin Resistant *S. aureus* ST544], VISA [Vancomycin Intermediate *S. aureus* 35403], *S. aureus* NCIMB 9518) and *Listeria monocytogenes* F2365] and against the yeasts *Candida* spp. [*C. glabrata* CBS138 and *C. albicans* MY1055] and the fungus *Aspergillus fumigatus* ATCC 46645 test strains using a deferred antagonism assay [43]. It should be noted that not all isolates were tested against all test strains. Only the results shown in Table 1 were performed. Isolates were spotted to the centre of Petri dishes on SYP-SW agar and grown until the colony reached 1–2 cm in diameter. Test strains were grown overnight in 5 mL LB broth, shaking, 200 rpm until they reached an OD600_nm_ 0.8. Test strains were then diluted 1:50 in LB soft agar (0.7% agar—*w*/*v*). Inoculated soft agar was poured onto the surface of the plates containing the isolates. Plates were incubated at 37 °C and examined the next day for zones of inhibition—clearance zones in the growth of the test strain around the colony of the isolate. 

### 3.7. Whole Genome Sequencing

For whole genome sequencing, mate pair libraries were prepared the using the Nextera XT DNA Library Preparation Kit (Illumina, San Diego, CA, USA) according to the manufacturer’s instructions. All libraries were sequenced in 250 bp paired read runs on the Illumina MiSeq platform. Reads were trimmed for quality with Sickle (Available at https://github.com/najoshi/sickle) and Scythe (Available at https://github.com/vsbuffalo/scythe).

### 3.8. Bioinformatic Methods

Genome assemblies were performed using SPAdes, version 3.1.1 using K-mer lengths of 21, 33, 55, 77, 81 and 91 [44]. Pan genome analysis was performed using Get Homologues [45]. Comparative genomic methods were carried out on the following 25 genomes which included the 13 isolates from Irish marine sponges presented here and the following 12 terrestrial *Streptomyces* spp. genomes from the sequence databases: *S. albus* J1074, *S. avermitilis* MA46-80, *S. bingchenggensis* BCW-1, *S. coelicolor* A3(2), *S. davaoensis* JCM 4913, *S. fulvissimus* DSM 40593, *S. hygroscopicus* subsp *jingganggensis* 5008, *S. griseus* NBRC 13350, *S. lividans*, *S. sirex* AA-E, *S. venezuelae* ATCC 10712 and *S. violaceusniger* Tü4113. Potential bioactive gene clusters were identified by searching the genomes with antiSMASH version 3 and parsed with R methods, R is a free software environment for statistical computing and graphics (The R Project―available at https://www.r-project.org/). Pfam domains often represented within these gene clusters were counted and graphed using R.

## 4. Conclusions

Analysis of the draft genomes of 11 *Streptomyces* spp. isolated from a shallow water sponge and an additional two isolated from deep sea sponges reveal the presence of highly abundant smBGCs. Amongst the 485 gene clusters identified, the majority display little or no homology with known smBGCs in the MIBiG database. The notably abundant PKS, NRPS, PKS/NRPS hybrid, bacteriocin and lantipeptide gene clusters are of particular interest in the search for novel antibiotic families and species in light of the worrisome trends in the occurrences of antimicrobial resistant human pathogens, particularly so because many of these strains have effectively inhibited the growth of problematic pathogenic strains (hVISA, MRSA) in plate screens. 

*Availability of data and material*: The datasets generated during and/or analysed during the current study are available in GenBank hosted by NCBI under the BioProject accession PRJNA384120 (https://www.ncbi.nlm.nih.gov/bioproject/PRJNA384120/) with individual genome accession codes NETW00000000, NETX00000000, NETY00000000, NETZ00000000, NEUA00000000, NEUB00000000, NEUC00000000, NEUD00000000, NEUE00000000, NEUF00000000, NEUG00000000, NEUH00000000, NEUI00000000.

## Figures and Tables

**Table 1:** Antimicrobial activities of cultured sponge bacteria using deferred antagonism assays. ^1^ *Bacillus cereus* FPL1; ^2^ *Bacillus subtilis* 1A40; ^3^ *B. subtilis* 1E32; ^a^ hVISA (Heterogonous Vancomycin Intermediate *Staphylococcus aureus*) 22900; ^b^ MRSA (Methicillin resistant *S. aureus*) ST544; ^C^ VISA (Vancomycin intermediate *S. aureus*) 35403; ^d^ *S. aureus* NCIMB 9518: ‘+’ = positive. ‘−’ = negative; n.d. not determined. * adapted from Kennedy et al., 2009 [19].

Test Strain	Gram Negative Bacteria		Gram Positive Bacteria			Yeasts	
	*E. coli* NCIMB 12210	*P. aeruginosa* PAO1	*Bacillus* pp.	*Staphylococcus* spp.	*L. monocyte genes* F2365	*Candida* spp.	*A. fumigatus* ATCC 46645
SM1 *	+	−	+ ^1,2^	+ ^a^	+	+	n.d.
SM5 *	+	−	+ ^1,2^	+ ^a^	−	−	n.d.
SM9 *	+	−	−	−	−	−	n.d.
SM10 *	+	n.d.	−	−	−	−	n.d.
SM11 *	−	−	+ ^2^	+ ^a,b^	−	−	n.d.
SM12 *	−	+	−	+ ^a^	−	−	n.d.
SM14 *	−	−	+ ^1,2^	+ ^a^	−	−	n.d.
SM16 *	−	+	+ ^2^	+ ^c^	−	−	n.d.
SM17 *	+	−	−	+ ^b^	−	+	n.d.
SM18 *	−	−	+ ^2^	+ ^b^	−	−	n.d.
FMC008 *	−	+	+ ^2^	+ ^d^	−	−	n.d.
B226SN101	−	−	− ^3^	−	n.d.	+	+
B188M101	−	−	− ^3^	−	n.d.	+	+

**Table 2:** No. of contigs and total genome size of marine *Streptomyces* spp. isolates.

Isolate ID	No. of Contigs	Total Length (Mb)
B188SM101	609	8.23
B226SN101	580	8.39
SM5	469	7.62
SM10	195	7.48
SM11	311	8
SM12	910	6.5
SM16	388	8.44
SM1	1057	8.08
SM9	1592	6.47
FMC008	1369	6.5
SM18	403	7.6
SM14	639	6.41
SM17	674	7.106

**Table 3:** Numbers of secondary metabolism gene clusters in the genomes of marine *Streptomyces* spp. isolates. * Numbers of PKS “types” (I, II & III) can be seen in Supplementary Tables S2–S14.

Isolate ID	PKS *	NRPS	PKS/NRPS Hybrid	Bacteriocin	Lantipeptide	Siderophore	Terpene	Butyrolactone	Ectoine	Other	TOTAL
B188M101	4	19	4	2	4	2	5	2	2	9	53
B226SN101	2	20	4	2	3	2	5	2	2	9	51
SM5	5	9	5	2	1	1	6	1	1	7	38
SM10	16	3	3	5	3	2	4	2	1	5	44
SM11	10	11	5	2	3	2	6	2	2	11	54
SM12	18	7	-	1	3	4	1	1	1	4	40
SM16	2	5	6	1	5	2	7	2	2	7	39
SM1	4	8	1	2	2	2	4	1	-	5	28
SM9	3	2	-	1	-	2	6	-	1	1	16
FMC008	1	3	-	1	-	1	5	-	1	3	15
SM18	9	9	6	2	2	1	6	1	1	4	41
SM14	8	2	-	-	1	2	-	1	-	3	17
SM17	14	18	2	3	2	3	5	-	1	1	49

## Supplementary Materials

The following are available online at http://www.mdpi.com/1660-3397/16/2/67/s1, Figure S1: Phylogenetic tree of deduced amino acid sequences of KS domains from PKS gene clusters from *Streptomyces* spp.; Figure S2: Phylogenetic tree of deduced amino acid sequences of condensation starter domains of NRPS gene clusters from *Streptomyces* spp.; Figure S3: Phylogenetic tree of deduced amino acid sequences of condensation DCL domains NRPS gene clusters from *Streptomyces* spp.; Figure S4: Phylogenetic tree of deduced amino acid sequences of condensation LCL domains from NRPS gene clusters from *Streptomyces* spp.; Figure S5: Phylogenetic tree of deduced amino acid sequences of (A) epimerization domains and (B) heterocyclization domains from NRPS gene clusters from *Streptomyces* spp.; Figure S6: Phylogenetic tree of deduced amino acid sequences of IucA-IucC domains from PKS gene clusters from *Streptomyces* spp.; Figure S7: Phylogenetic tree of deduced amino acid sequences of DUF692 domains from bacteriocin gene clusters from *Streptomyces* spp.; Figure S8: Phylogenetic tree of deduced amino acid sequences of LanC-like domains from lantipeptide gene clusters from *Streptomyces* spp.; Table S1: Taxonomy of *Streptomyces* spp. isolates using KRAKEN; Table S2: Secondary metabolism biosynthetic gene clusters in the genome of *Streptomyces* sp. B188M101 as predicted by antiSMASH; Table S3: Secondary metabolism biosynthetic gene clusters in the genome of *Streptomyces* sp. B226SN101 as predicted by antiSMASH; Table S4: Secondary metabolism biosynthetic gene clusters in the genome of *Streptomyces* sp. SM5 as predicted by antiSMASH; Table S5: Secondary metabolism biosynthetic gene clusters in the genome of *Streptomyces* sp. SM10 as predicted by antiSMASH; Table S6: Secondary metabolism biosynthetic gene clusters in the genome of *Streptomyces* sp. SM11 as predicted by antiSMASH; Table S7: Secondary metabolism biosynthetic gene clusters in the genome of *Streptomyces* sp. SM12 as predicted by antiSMASH; Table S8: Secondary metabolism biosynthetic gene clusters in the genome of *Streptomyces* sp. SM16 as predicted by antiSMASH; Table S9: Secondary metabolism biosynthetic gene clusters in the genome of *Streptomyces* sp. SM17 as predicted by antiSMASH; Table S10: Secondary metabolism biosynthetic gene clusters in the genome of *Streptomyces* sp. SM18 as predicted by antiSMASH; Table S11: Secondary metabolism biosynthetic gene clusters in the genome of *Streptomyces* sp. SM1 as predicted by antiSMASH; Table S12: Secondary metabolism biosynthetic gene clusters in the genome of *Streptomyces* sp. FMC008 as predicted by antiSMASH; Table S13: Secondary metabolism biosynthetic gene clusters in the genome of *Streptomyces* sp. SM14 as predicted by antiSMASH; Table S14: Secondary metabolism biosynthetic gene clusters in the genome of *Streptomyces* sp. SM9 as predicted by antiSMASH.
