# Supplementary material for: Diverse and Abundant Secondary Metabolism Biosynthetic Gene Clusters in the Genomes of Marine Sponge Derived Streptomyces spp. Isolates
Source: Mar Drugs. 2018 Feb 20;16(2):67. doi: 10.3390/md16020067 (PMC5852495; doi:10.3390/md16020067)
Supplement: Supplementary file 1 [file marinedrugs-16-00067-s001.pdf]

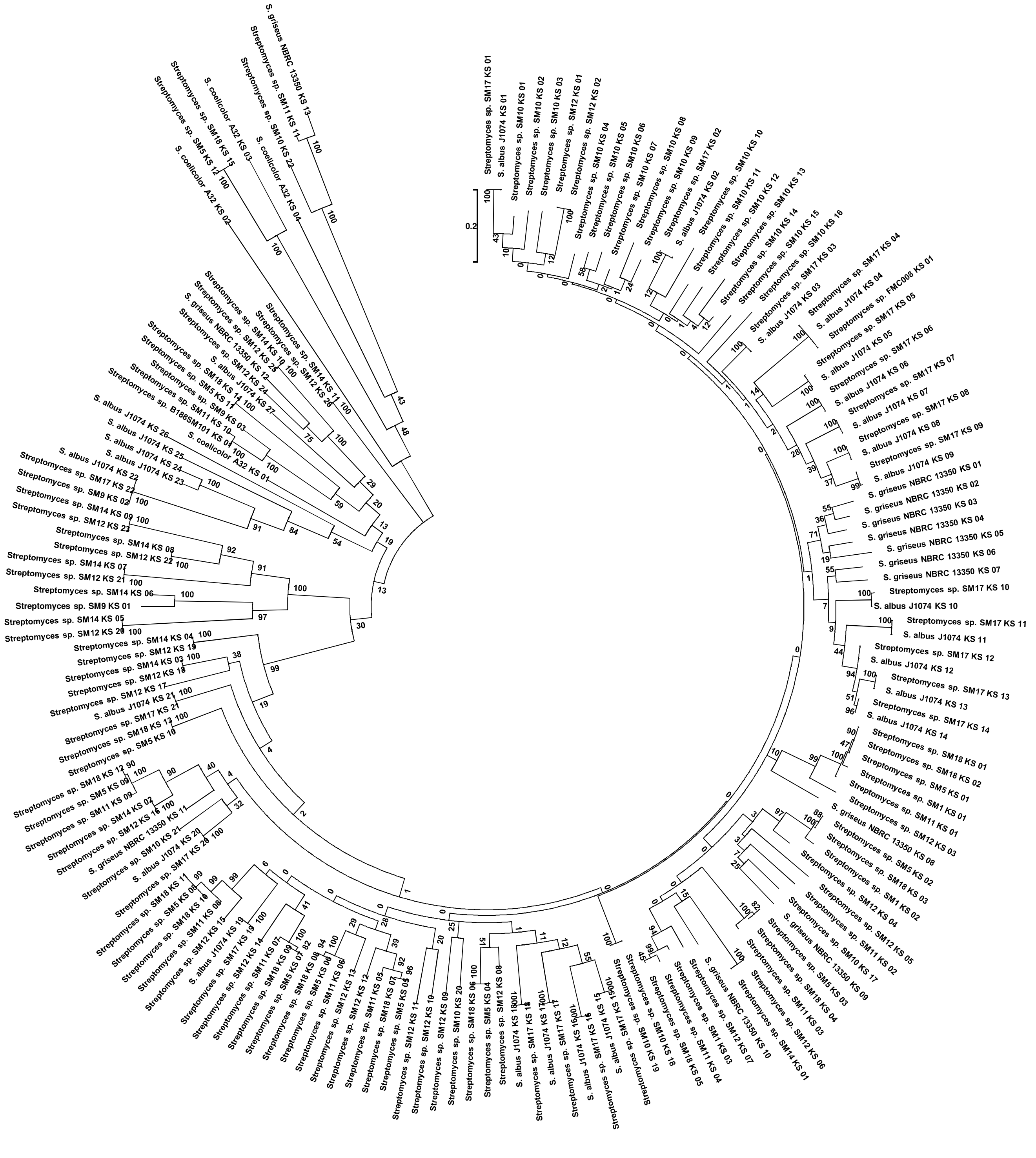

Supplementary Figure S1. Bootstrap-consensus (n=100) Maximum Likelihood phylogenetic tree of KS domain, deduced amino acid sequences of PKS gene clusters, from the genomes of marine *Streptomyces* spp. and from the genomes of selected reference terrestrial *Streptomyces* spp.

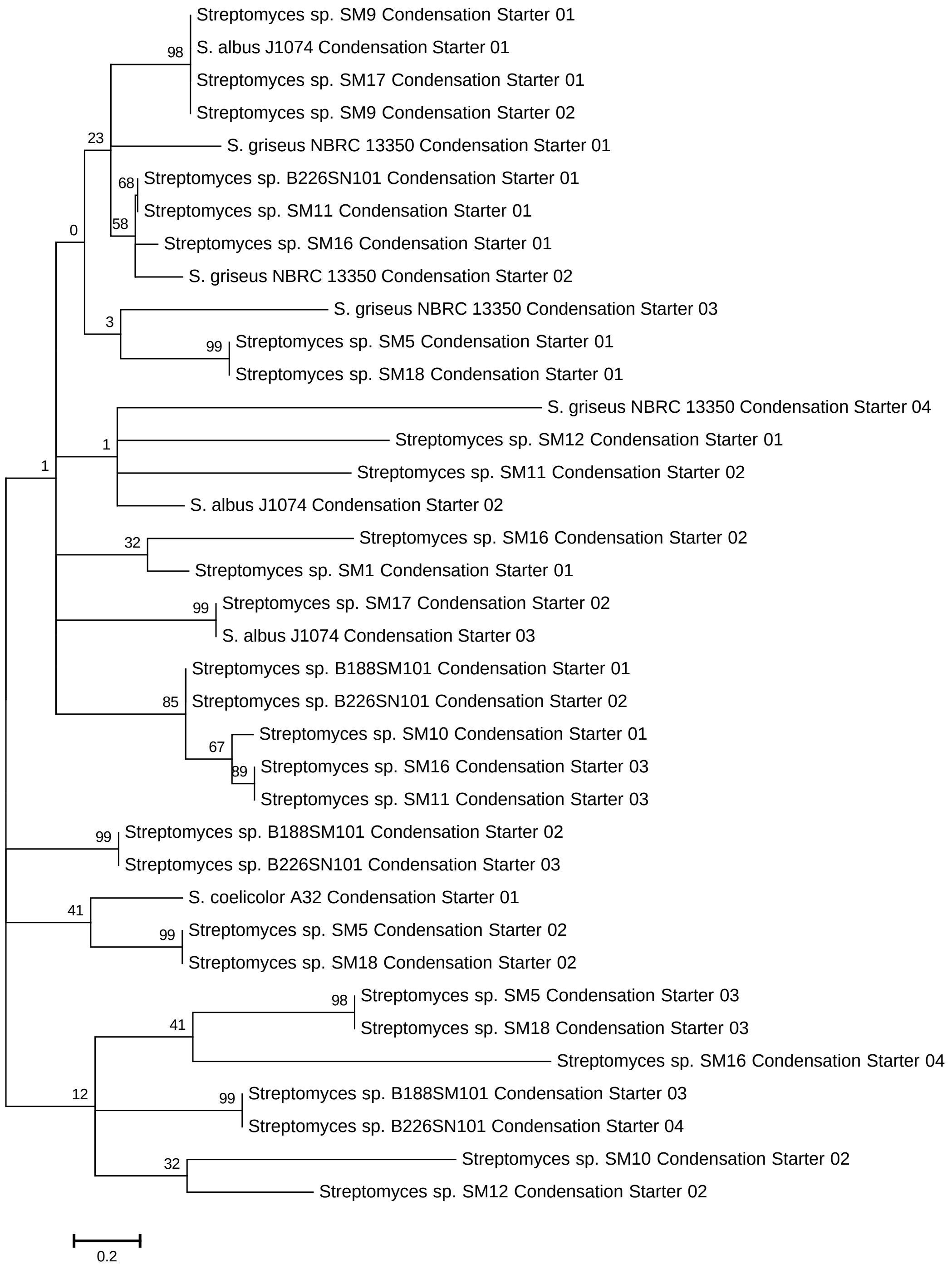

Supplementary Figure S2. Bootstrap-consensus (n=100) Maximum Likelihood phylogenetic tree of condensation starter domain, deduced amino acid sequences, of NRPS gene clusters, from the genomes of marine Streptomyces spp. and from the genomes of selected reference terrestrial Streptomyces spp.

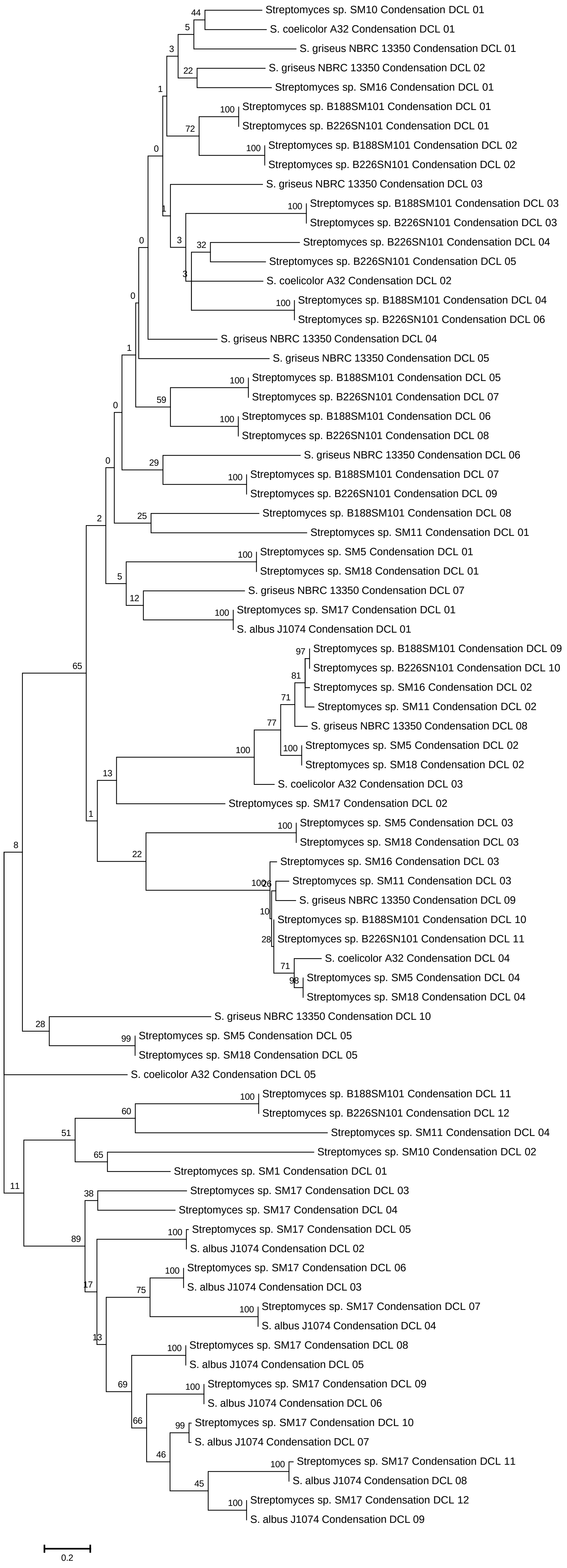

Supplementary Figure S3. Bootstrap-consensus (n=100) Maximum Likelihood phylogenetic tree of condensation DCL domain, deduced amino acid sequences, of NRPS gene clusters, from the genomes of marine *Streptomyces* spp. and from the genomes of selected reference terrestrial *Streptomyces* spp.

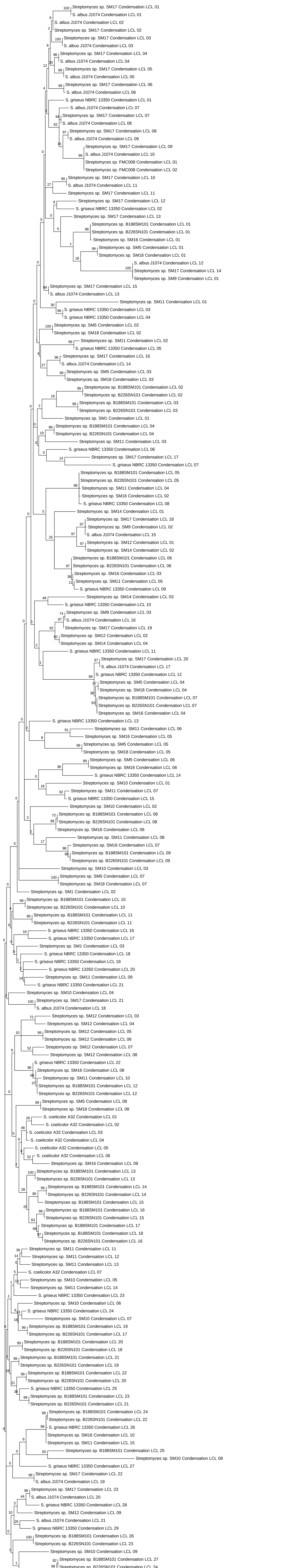

Supplementary Figure S4. Bootstrap-consensus (n=100) Maximum Likelihood phylogenetic tree of condensation LCL domain, deduced amino acid sequences, of NRPS gene clusters from the genomes of marine Streptomyces spp. and from the genomes of selected reference terrestrial Streptomyces spp.

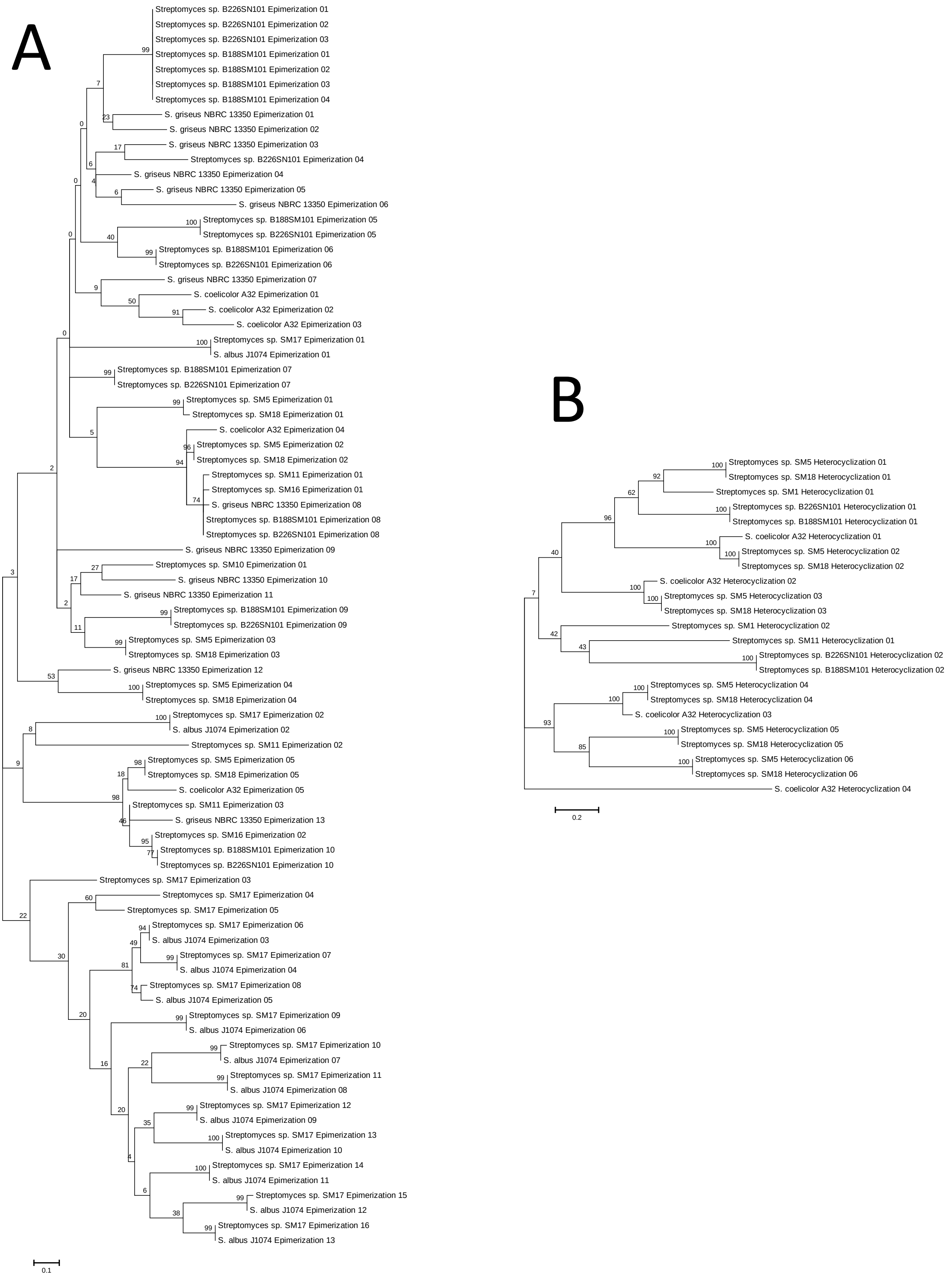

Supplementary Figure S5. Bootstrap-consensus (n=100) Maximum Likelihood phylogenetic trees of deduced amino acid sequences of (A) epimerization and (B) heterocyclization domains from NRPS gene clusters, from the genomes of marine Streptomyces spp. and from the genomes of selected reference terrestrial Streptomyces spp.

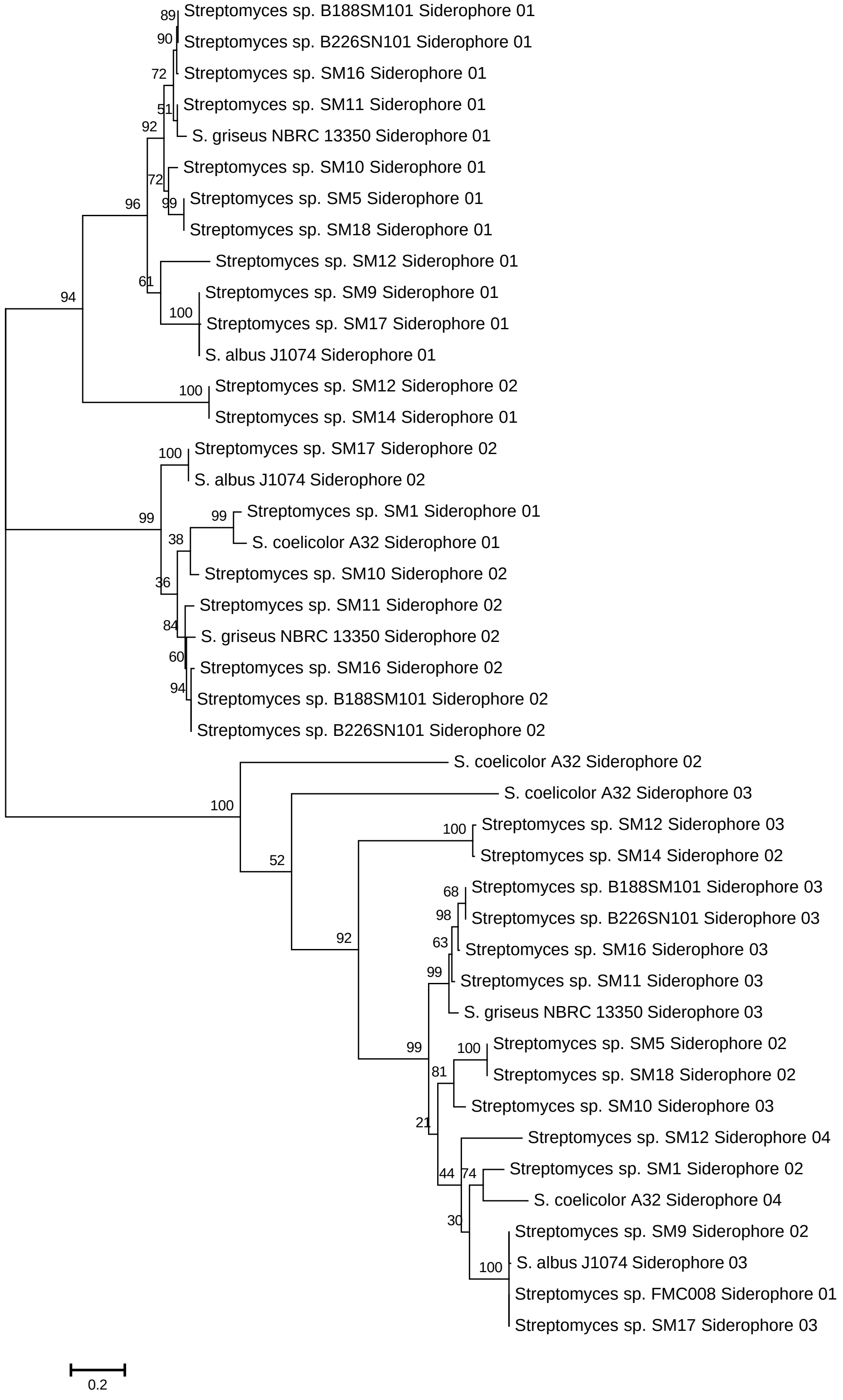

Supplementary Figure S6. Bootstrap-consensus (n=100) Maximum Likelihood phylogenetic trees of deduced amino acid sequences of IucA-IucC domains from siderophore gene clusters, from the genomes of marine Streptomyces spp. and from the genomes of selected reference terrestrial Streptomyces spp.

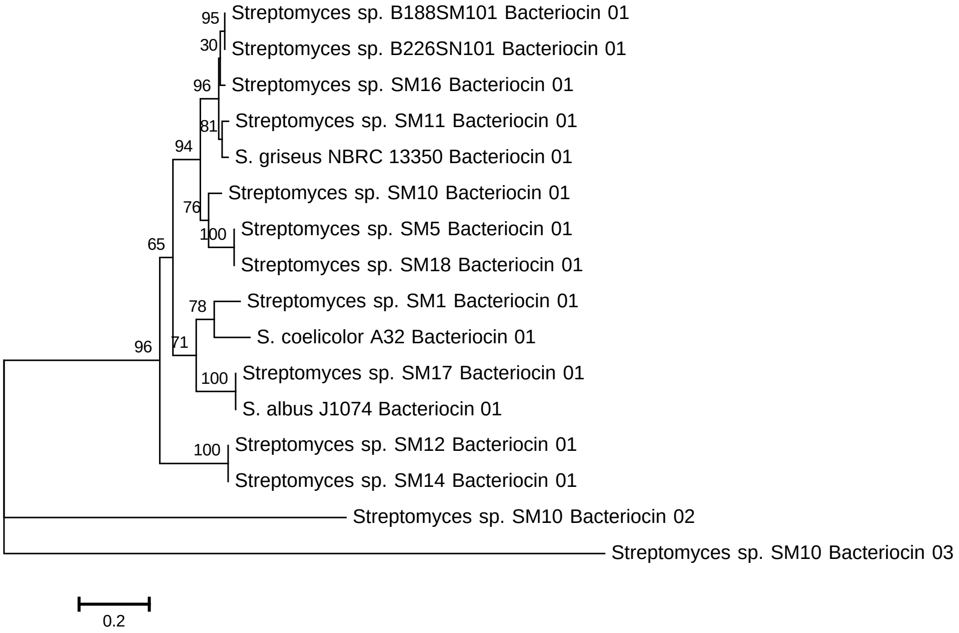

Supplementary Figure S7. Bootstrap-consensus (n=100) Maximum Likelihood phylogenetic trees of deduced amino acid sequences of DUF692 domains from bacteriocin gene clusters, from the genomes of marine Streptomyces spp. and from the genomes of selected reference terrestrial Streptomyces spp.

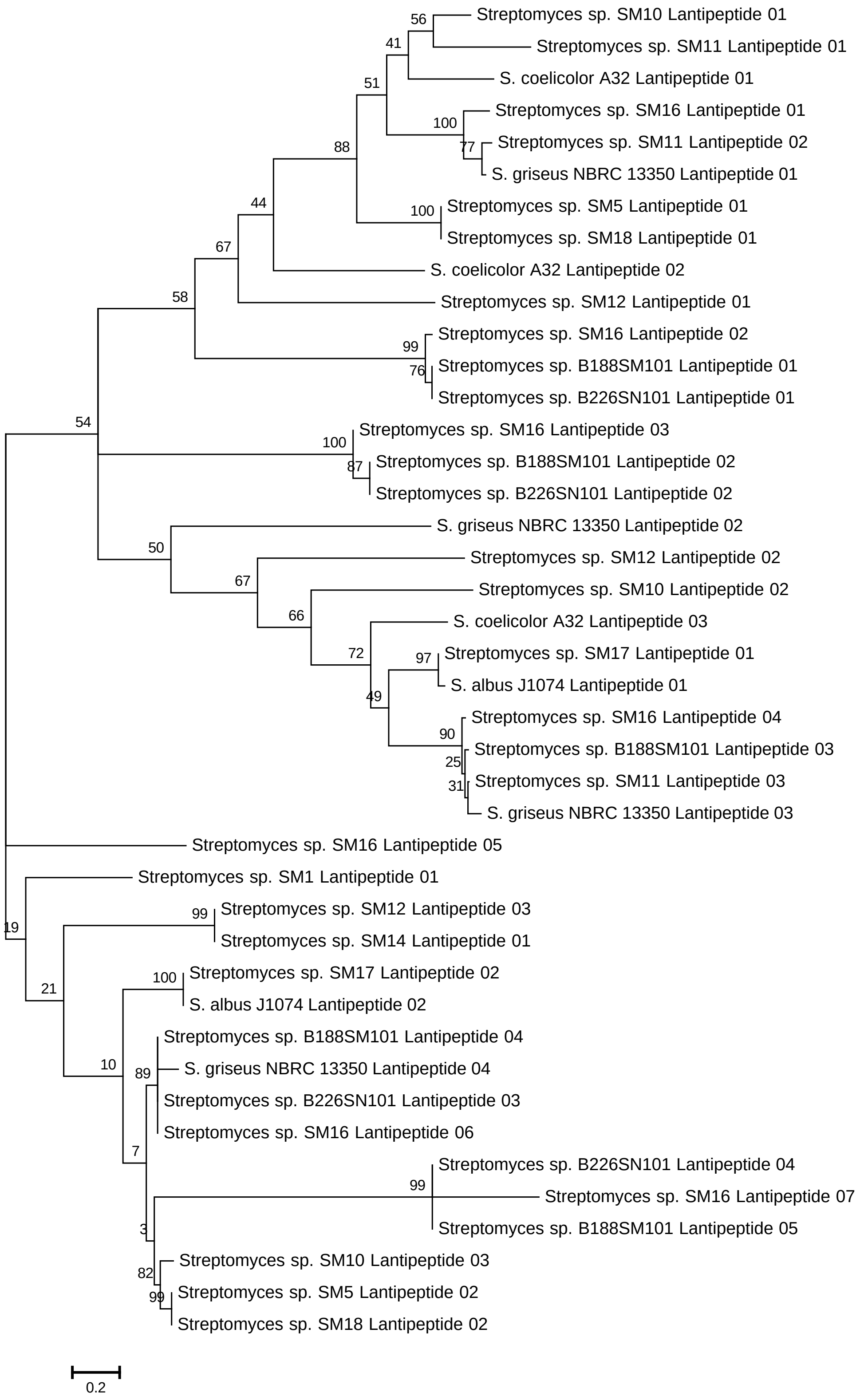

Supplementary Figure S8. Bootstrap-consensus (n=100) Maximum Likelihood phylogenetic trees of deduced amino acid sequences of LanC-like domains from lantipeptide gene clusters, from the genomes of marine Streptomyces spp. and from the genomes of selected reference terrestrial Streptomyces spp.

Isolate ID	Percentage of sequences assigned to closest sequence in kraken classification with miniKraken database
SM1	31.7% *Streptomyces coelicolor* A3 (2)<br>18.1% *Streptomyces* root
SM5	83.91% *Streptomyces sp*. Sirex AA-E
SM9	97.4% *Streptomyces albus* J1074
SM10	55.5% *Streptomyces sp*. Sirex AA-E
SM11	78.7% *Streptomyces griseus subsp. griseus* NBRC 13350
SM12	33.3% *Streptomyces* root<br>47.5% assigned to various *Streptomyces spp*.
SM14	35.1% *Streptomyces* root<br>47.4% assigned to various *Streptomyces spp*.
SM16	80.45% *Streptomyces griseus subsp. griseus* NBRC 13350
SM17	97.8% *Streptomyces albus* J1074
SM18	84.5% *Streptomyces sp.* Sirex AA-E
FMC008	97.2% *Streptomyces albus* J1074
B188M101	82.7% *Streptomyces griseus subsp. griseus* NBRC 13350
B226SN101	82.22% *Streptomyces griseus subsp. griseus* NBRC 13350

Supplementary Table S1. Taxonomy of *Streptomyces* spp. isolates using KRAKEN.

	B188M101		
Cluster no.	Cluster type	Most similar known cluster	% of genes showing similarity
1	NRPS	Heat Stable Antifungal Factor (HSAF)	62
2	Butyrolactone	Gamma - butyroactone	100
3	Siderophore	Desferrioxamine B	100
4	NRPS-T1PKS	Balhimycin	8
5	NRPS	none	0
6	Lassopeptide	SRO15-2005	80
7	Others KS	none	0
8	NRPS	Calcium-dependent antibiotic	5
9	Terpene	none	0
10	Ladderane-Arylpoyene	Skyllamycin	12
11	Melanin	Melanin	100
12	Ectoine	Ectoine	100
13	Bacteriocin	none	0
14	Lantipeptide	none	0
15	T3-PKS	Alkylresorcinol	66
16	Bacteriocin	none	0
17	Others-Nrps-T1PKS	Zorbamycin	4
18	T1PKS-NRPS	none	0
19	NRPS	Thaxtomin	18
20	Terpene	none	0
21	Other	none	0
22	T1PKS	SGR-PTMs	33
23	NRPS	none	0
24	T3PKS	Herboxidiene	6
25	NRPS	none	0
26	Terpene	none	0
27	NRPS	Laspartomycin	6
28	NRPS	none	0
29	NRPS	Daptomycin	18
30	Lantipeptide	none	0
31	Phenazine	Lomofungin	26
32	NRPS	none	0
33	NRPS	Friulimicin	6
34	NRPS	Skyllamycin	16
35	Terpene	Hopene	69
36	Siderophore	Kinamycin	8
37	NRPS	Friulimicin	15
38	Butyrolactone	Skyllamycin	14
39	Ectoine	Kosinostatin	11
40	Lantipeptide	AmfS	60
41	NRPS	none	0
42	Thiopeptide-Lantipeptide	none	0
43	NRPS-OthersKS	Kirromycin	8
44	Lassopeptide	Chlortetracycline	5
45	NRPS	Griseobactin	52
46	Lantipeptide	Chalcomycin	9
47	NRPS	none	0
48	Terpene	Isorenieratene	100
49	NRPS	Friulimicin	18
50	NRPS	Coelichelin	72
51	NRPS	Daptomycin	4
52	NRPS	none	0
53	NRPS	none	0

Supplementary Table S2. Secondary metabolism gene cluster types in the draft genome of B188M101, most similar known clusters and % of genes showing similarity to known clusters as determined by antiSMASH.

	B226SN101		
Cluster no.	Cluster type	Most similar known cluster	% of genes showing similarity
1	Bacteriocin	none	0
2	Terpene	none	0
3	Others KS	Eicosapentanoic acid	33
4	Arylpolyene-Ladderene	Skyllamycin	12
5	NRPS-T1PKS	HSAF	75
6	Butyrolactone	Gamma Butyrolactone	100
7	NRPS	none	0
8	Siderophore	Desferrioxamine B	100
9	Melanin	Melanin	100
10	Terpene	none	0
11	NRPS-T1PKS	Zorbamycin	4
12	Ectoine	Kosinostatin	11
13	Lantipeptide	none	0
14	Bacteriocin	none	0
15	T3PKS	Herboxidiene	6
16	Other	none	0
17	NRPS	none	0
18	NRPS	none	0
19	NRPS	none	0
20	Terpene	Hopene	69
21	NRPS	Calcium-dependent antibiotic	7
22	Terpene	none	0
23	NRPS	Friulimicin	15
24	NRPS	none	0
25	Phenazine	Lomofungin	26
26	NRPS	Griseobactin	29
27	Lassopeptide	SRO15-2005	60
28	Lassopeptide	none	0
29	NRPS	none	0
30	Siderophore	Kinamycin	8
31	NRPS	Daptomycin	18
32	Lantipeptide	none	0
33	NRPS	none	0
34	NRPS	none	0
35	NRPS-T1PKS	Kanamycin	2
36	NRPS	Virginiamycin	11
37	Lantipeptide	Guadinomine	7
38	NRPS	Friulimicin	15
39	NRPS-Others KS	Kirromycin	8
40	Butyrolactone	Skyllamycin	14
41	Ectoine	Ectoine	100
42	Thiopeptide-Lantipeptide	none	0
43	T3PKS	Alkylresorcinol	100
44	NRPS	Grisobactin	64
45	Terpene	Isorenieratene	100
46	NRPS	Coelichelin	72
47	NRPS	none	0
48	NRPS	Daptomycin	4
49	NRPS	WS9326	22
50	NRPS	Friulimicin	6
51	NRPS	none	0

Supplementary Table S3. Secondary metabolism gene cluster types in the draft genome of B226SN101, most similar known clusters and % of genes showing similarity to known clusters as determined by antiSMASH.

	SM5		
Cluster no.	Cluster type	Most similar known cluster	% of genes showing similarity
1	Others-T1PKS	none	0
2	Others-KS	none	0
3	Terpene	Isorenieratene	42
4	Other	Gobichelin	11
5	NRPS	Coelichelin	72
6	Bacteriocin	none	0
7	NRPS	Coelichelin	27
8	NRPS	none	0
9	Terpene	Carbapenem_MM_4550	6
10	Terpene	Steffimycin	11
11	Thiopeptide-Lantipeptide	none	0
12	NRPS	none	0
13	Ectoine	Ectoine	100
14	Others-KS	A33853	34
15	T1PKS	Lasalocid	5
16	Butyrolactone	Lactonamycin	5
17	Terpene	none	0
18	T1PKS	Surfactin	8
19	Bacteriocin	none	0
20	NRPS	Mirubactin	50
21	Lantipeptide-Lassopeptide	none	0
22	T1PKS	Bafilomycin	33
23	NRPS	Paenibactin	83
24	NRPS-T1PKS	Herboxidiene	15
25	Siderophore	Kinamycin	11
26	T1PKS-NRPS	Myxothiazol	42
27	Melanin	Istamycin	2
28	NRPS	Griseoviridin/Viridogrisien	2
29	Terpene	Carotenoid	27
30	T1PKS	Bafilomycin	83
31	Other KS	none	0
32	T2PKS-Terpene	Spore Pigment	75
33	NRPS	Coelibactin	90
34	Terpene	Hopene	69
35	T1PKS	Indanomycin	17
36	Lantipeptide	none	0
37	T2PKS-Oligosaccharide	Pristinamycin	25
38	NRPS	SW163	28

Supplementary Table S4. Secondary metabolism gene cluster types in the draft genome of SM5, most similar known clusters and % of genes showing similarity to known clusters as determined by antiSMASH.

	SM10		
Cluster no.	Cluster type	Most similar known cluster	% of genes showing similarity
1	Beta-Lactam	Clavams	35
2	Ectoine	Ectoine	100
3	T1PKS	none	0
4	T1PKS	Tetronomycin	18
5	T1PKS	ECO-02301	39
6	Others-T2PKS	Cinerubin B	28
7	T1PKS	Micromonolactam	100
8	T1PKS	none	0
9	Siderophore	Desferrioxamine B	83
10	Terpene-T2PKS	Spore Pigment	83
11	Melanin	Istamycin	4
12	NRPS	Cystathiazole A	11
13	T3PKS	Tetronasin	11
14	T2PKS	Jadomycin	100
15	Lantipeptide	none	0
16	T1PKS	Lasalocid	13
17	Terpene	none	0
18	Siderophore	none	0
19	Bacteriocin	none	0
20	Butyrolactone	Zorbamycin	6
21	Terpene	Steffinycin	19
22	Lantipeptide	Fluostatin	4
23	T1PKS	none	0
24	T1PKS	none	0
25	Terpene	none	0
26	T1PKS	Sporolide	21
27	Bacteriocin	none	0
28	T1PKS	Aculeximycin	23
29	NRPS	Daptomycin	9
30	Bacteriocin	none	0
31	Thiopeptide	none	0
32	Bacteriocin	none	0
33	T1PKS-Bacteriocin	Hygrocin	29
34	Terpene	Hopene	76
35	Lantipeptide	Ansatrienin (mycotrienin)	7
36	NRPS	Tetronasin	3
37	Butyrolactone	Rabelomycin	12
38	Bacteriocin	none	0
39	Other	Laspartomycin	11
40	Oligosaccharide	none	0
41	T1PKS	Ebelactone	50
42	T1PKS	Thuggacin	15
43	T1PKS	none	0
44	T1PKS	none	0

Supplementary Table S5. Secondary metabolism gene cluster types in the draft genome of SM10, most similar known clusters and % of genes showing similarity to known clusters as determined by antiSMASH.

	SM11		
Cluster no.	Cluster type	Most similar known cluster	% of genes showing similarity
1	Lassopeptide	none	0
2	NRPS	Coelichelin	81
3	Other	none	0
4	Melanin	Istamycin	4
5	Terpene	Steffimycin	11
6	Other	none	0
7	T1PKS	Bafilomycin	44
8	Lantipeptide	AmfS	100
9	Ectoine	Ectoine	75
10	Thiopeptide-Lantipeptide	none	0
11	NRPS	none	0
12	NRPS	none	0
13	Lantipeptide	none	0
14	T1PKS	Bafilomycin	83
15	T1PKS	Concanamycin A	21
16	NRPS	none	0
17	T3PKS	Alkylresorcinol	100
18	T1PKS	C-1027	18
19	NRPS	A47934	8
20	Terpene-Others KS	Pristinamycin	6
21	Ectoine	Kosinostatin	11
22	Butyrolactone	none	0
23	Terpene	Platensimycin/Platencin	8
24	Terpene	Isorenieratene	100
25	T1PKS-NRPS	none	0
26	NRPS	Daptomycin	7
27	T1PKS	SGR-PTMs	66
28	T2PKS	Granaticin	21
29	NRPS	Streptolygidin	5
30	Lantipeptide	none	0
31	Other	none	0
32	NRPS	Tetronasin	3
33	T3PKS	Naringenin	100
34	T1PKS	none	0
35	Other KS	Arsenopolyketides	20
36	NRPS-T1PKS	Herboxidiene	4
37	NRPS	Kirromycin	28
38	Trans AT PKS-NRPS-T1PKS	Kirromycin	35
39	Terpene	none	0
40	Lassopeptide	SRO15-2005	80
41	Terpene	Hopene	69
42	Melanin	none	0
43	Bacteriocin	none	0
44	Siderophore	Desferrioxamine B	100
45	Siderophore	none	0
46	NRPS	Friulimicin	21
47	Other	Roseoflavin	100
48	NRPS	Griseobactin	58
49	Terpene	none	0
50	Butyrolactone	Gamma Butyrolactone	100
51	NRPS-Bacteriocin	C-1027	63
52	T2PKS	Chartreusin	33
53	Bacteriocin	none	0
54	Other	Actinomycin	7

Supplementary Table S6. Secondary metabolism gene cluster types in the draft genome of SM11, most similar known clusters and % of genes showing similarity to known clusters as determined by antiSMASH.

	SM12		
**Cluster no.**	**Cluster type**	**Most similar known cluster**	**% of genes showing similarity**
1	T1PKS	Stambomycin	52
2	Bacteriocin	none	0
3	T1PKS	none	0
4	T1PKS	none	0
5	T1PKS	Guadinomine	7
6	T1PKS	none	0
7	NRPS	none	0
8	Lantipeptide	none	0
9	Other KS	none	0
10	Oligosaccharide	Pellasoren	16
11	T1PKS	none	0
12	T1PKS	none	0
13	Siderophore	none	0
14	T1PKS	none	0
15	Other KS	none	0
16	NRPS	Laspartomycin	6
17	Lantipeptide	none	0
18	T1PKS	none	0
19	Siderophore	none	0
20	Terpene	none	0
21	T1PKS	none	0
22	Lantipeptide	Kanamycin	3
23	T1PKS	Stambomycin	60
24	T1PKS	Nigericin	55
25	Butyrolactone	none	0
26	NRPS	none	0
27	T1PKS	none	0
28	Ectoine	Ectoine	100
29	T1PKS	Concanamycin	21
30	T3PKS	Zorbamycin	4
31	Siderophore	none	0
32	Siderophore	none	0
33	T1PKS	Sanglifehrin A	6
34	NRPS	Marfomycins	8
35	T1PKS	Thuggacin	15
36	NRPS	none	0
37	NRPS	none	0
38	NRPS	none	0
39	B-Lactam	Clavulanic acid	20
40	T1PKS	Concanamycin A	28

Supplementary Table S7. Secondary metabolism gene cluster types in the draft genome of SM12, most similar known clusters and % of genes showing similarity to known clusters as determined by antiSMASH.

	SM16		
Cluster no.	Cluster type	Most similar known cluster	% of genes showing similarity
1	Terpene	Steffimycin	11
2	Lantipeptide	AmfS	40
3	Ectoine	Kosinostatin	9
4	Thiopeptide-Lantipeptide	none	0
5	Siderophore	Desferrioxamine B	100
6	NRPS	Oxazolomycin	6
7	Other	none	0
8	T3PKS	Alkylresorcinol	100
9	Terpene	Hopene	69
10	NRPS-T1PKS	Kanamycin	2
11	Bacteriocin-NRPS	Tetronasin	7
12	NRPS-T1PKS	Heat Stable Antifungal Factor (HSAF)	75
13	NRPS	none	0
14	Others KS-NRPS	Bacillibactin	15
15	Lantipeptide	Guadinomine	7
16	Terpene	Glycopeptidolipid	20
17	NRPS-T1PKS-Lantipeptide	none	0
18	Bacteriocin	none	0
19	T3PKS	Herboxidiene	6
20	Lantipeptide	none	0
21	Lassopeptide	SRO15-2015	80
22	Melanin	Melanin	100
23	Ectoine	Ectoine	100
24	Terpene	none	0
25	Terpene	none	0
26	Terpene	Isorenieratene	85
27	Other KS	Borrelidin	9
28	Other	BE-14106	10
29	Other KS-T1PKS-Terpene	Isorenieratene	85
30	Lantipeptide	SRO15-2015	100
31	Lantipeptide	Labyrinthopeptin	40
32	Butyrolactone	Gamma-Butyrolactone	100
33	Terpene	none	0
34	NRPS	Griseobactin	94
35	NRPS	Coelichelin	81
36	Siderophore	Kinamycin	11
37	NRPS	C-1027	37
38	T1PKS-Butyrolactone	Neocarzinostatin	52
39	NRPS	none	0

Supplementary Table S8. Secondary metabolism gene cluster types in the draft genome of SM16, most similar known clusters and % of genes showing similarity to known clusters as determined by antiSMASH.

	SM17		
Cluster no.	Cluster type	Most similar known cluster	% of genes showing similarity
1	NRPS	none	0
2	Terpene	none	0
3	NRPS	none	0
4	NRPS	none	0
5	T1PKS	none	0
6	Terpene	Hopene	46
7	Lantipeptide	none	0
8	Bacteriocin	Carotenoid	18
9	NRPS	Mannopeptimycin	40
10	Other	Albachelin	20
11	Lantipeptide	SAL-2242	40
12	NRPS	none	0
13	NRPS-T1PKS	SGR-PTMs	10
14	NRPS	none	0
15	NRPS	none	0
16	Ectoine	Ectoine	75
17	T1PKS	FR-008	71
18	Lantipeptide-NRPS-T1PKS	Antimycin	26
19	Siderophore	none	0
20	Bacteriocin	none	0
21	NRPS	none	0
22	Terpene	Albaflavenone	100
23	Terpene	none	0
24	Bacteriocin	none	0
25	T1PKS	none	0
26	T1PKS	none	0
27	T1PKS	none	0
28	T1PKS	Indanomycin	21
29	NRPS	Desotamide	9
30	NRPS	Tertronasin	9
31	Siderophore	Desferrioxamine B	100
32	NRPS	none	0
33	T3PKS	Herboxidiene	12
34	Terpene	none	0
35	T1PKS	none	0
36	NRPS	none	0
37	NRPS	none	0
38	NRPS	none	0
39	NRPS	none	0
40	T1PKS	ECO-02301	25
41	NRPS	none	0
42	NRPS	none	0
43	T1PKS	none	0
44	T1PKS	none	0
45	T1PKS	none	0
46	Siderophore	none	0
47	T1PKS	Halstoctacosanolide	77
48	NRPS	none	0

Supplementary Table S9. Secondary metabolism gene cluster types in the draft genome of SM17, most similar known clusters and % of genes showing similarity to known clusters as determined by antiSMASH.

	SM18		
Cluster no.	Cluster type	Most similar known cluster	% of genes showing similarity
1	Butyrolactone	Lactonamycin	5
2	Other KS	A33853	34
3	NRPS	Coelibactin	27
4	Terpene	Carotenoid	27
5	NRPS	Paenibactin	66
6	Melanin	Melanin	100
7	Lantipeptide	none	100
8	T1PKS	Lasalocid	5
9	Terpene	Steffimycin	13
10	Other KS	none	0
11	T1PKS	Kirromycin	3
12	NRPS	Coelichelin	27
13	NRPS	Coelichelin	72
14	Terpene	Isorenieratene	57
15	T1PKS-NRPS	Cystothiazole A	17
16	NRPS	none	0
17	Other KS-NRPS	SW-163	28
18	T1PKS	none	0
19	Other KS-T1PKS	none	0
20	NRPS	Mirubactin	50
21	Terpene	none	0
22	Siderophore	none	0
23	Lantipeptide-Lassopeptide	Lipopolysaccharide	5
24	T1PKS	Salinomycin	18
25	Terpene	Hopene	69
26	T1PKS	FD-891	62
27	NRPS-T1PKS	Herboxidiene	15
28	Oligsaccharide-T2PKS	Granaticin	43
29	Bacteriocin	none	0
30	Bacteriocin	none	0
31	T1PKS	Bafilomycin	83
32	NRPS	none	0
33	Ectoine	Ectoine	100
34	Terpene-T2PKS	Spore Pigment	75
35	Terpene	Carbapenem MM 4550	10
36	T1PKS	none	0
37	T1PKS	Bafilomycin	33
38	NRPS	Coelibactin	100
39	NRPS	Mannopeptimycin	7
40	T1PKS	none	0
41	Lantipeptide	none	0

Supplementary Table S10. Secondary metabolism gene cluster types in the draft genome of SM18, most similar known clusters and % of genes showing similarity to known clusters as determined by antiSMASH.

	SM1		
**Cluster no.**	**Cluster type**	**Most similar known cluster**	**% of genes showing similarity**
1	NRPS	none	0
2	Other KS	none	0
3	NRPS	none	0
4	NRPS	none	0
5	Lantipeptide	none	0
6	NRPS	Erythrochelin	42
7	Siderophore	Desferrioxamine B	100
8	Terpene	none	0
9	Siderophore	Kinamycin	8
10	NRPS	none	0
11	Terpene	Carotenoid	27
12	Lantipeptide	none	0
13	NRPS	none	0
14	T1PKS	Micromonolactam	100
15	T2PKS	Spore Pigment	83
16	Butyrolactone	none	0
17	Terpene	Isorenieratene	28
18	Terpene	Hopene	15
19	Other KS	none	0
20	Terpene	none	0
21	NRPS	none	0
22	Other	none	0
23	Bacteriocin	none	0
24	T1PKS	none	0
25	NRPS	none	0
26	T1PKS-NRPS	Myxalamid	45
27	T1PKS	none	0
28	Bacteriocin	none	0

Supplementary Table S11. Secondary metabolism gene cluster types in the draft genome of SM1, most similar known clusters and % of genes showing similarity to known clusters as determined by antiSMASH.

	FMC008		
**Cluster no.**	**Cluster type**	**Most similar known cluster**	**% of genes showing similarity**
1	Terpene	none	0
2	Ectoine	Ectoine	100
3	NRPS	Complestatin	25
4	Other	Aureothin	22
5	Terpene	none	0
6	Siderophore	none	0
7	Bacteriocin	none	0
8	T1PKS	none	0
9	Terpene	Phosphonoglycans	6
10	NRPS	Desotamide	13
11	Other	Antimycin	13
12	NRPS	none	0
13	Terpene	none	0
14	Other	none	0
15	Terpene	none	0

Supplementary Table S12. Secondary metabolism gene cluster types in the draft genome of FMC008, most similar known clusters and % of genes showing similarity to known clusters as determined by antiSMASH.

	SM14		
Cluster no.	Cluster type	Most similar known cluster	% of genes showing similarity
1	T1PKS	none	0
2	T1PKS	none	0
3	B-Lactam	Clavulanic acid	20
4	Siderophore	none	0
5	T1PKS	none	0
6	Lantipeptide	none	0
7	Oligosaccharide	Pellasoren	16
8	T1PKS	none	0
9	T1PKS	Aculeximycin	23
10	T1PKS	none	0
11	NRPS	Calium-dependent antibiotic	12
12	Thiopeptide=Lantipeptide	none	0
13	T3PKS	Zorbamycin	4
14	NRPS	Tetronasin	3
15	T1PKS	none	0
16	Siderophore	none	0
17	Bacteriocin	none	0

Supplementary Table S13. Secondary metabolism gene cluster types in the draft genome of SM14, most similar known clusters and % of genes showing similarity to known clusters as determined by antiSMASH.

	SM9		
Cluster no.	Cluster type	Most similar known cluster	% of genes showing similarity
1	Ectoine	Ectoine	100
2	Terpene	none	0
3	Siderophore	none	0
4	NRPS	Antimycin	20
5	T1PKS	Frontalamides	28
6	Terpene	Carotenoid	18
7	Terpene	none	0
8	Bacteriocin	none	0
9	Siderophore	none	0
10	Terpene	none	0
11	Other	none	0
12	Terpene	none	0
13	T1PKS	none	0
14	T1PKS	none	0
15	NRPS	Complestatin	25
16	Terpene	Hopene	15

Supplementary Table S14. Secondary metabolism gene cluster types in the draft genome of SM9, most similar known clusters and % of genes showing similarity to known clusters as determined by antiSMASH.
